# Airway-associated adipose tissue accumulation is increased in a kisspeptin receptor knockout mouse model

**DOI:** 10.1042/CS20230792

**Published:** 2023-10-03

**Authors:** Carolyn J. Wang, Jeremy T. Smith, David Lu, Peter B. Noble, Kimberley C.W. Wang

**Affiliations:** 1 School of Human Sciences, The University of Western Australia, Crawley, Western Australia, Australia; 2 Telethon Kids Institute, The University of Western Australia, Nedlands, Western Australia, Australia

**Keywords:** airway remodelling, airway-associated adipose tissue, Kiss1, Kiss1r, obesity

## Abstract

Airway-associated adipose tissue increases with body mass index and is a local source of pro-inflammatory adipokines that may contribute to airway pathology in asthma co-existing with obesity. Genetic susceptibility to airway adiposity was considered in the present study through kisspeptin/kisspeptin receptor signalling, known to modulate systemic adiposity and potentially drive airway remodelling. Therefore, the aim of the study was to determine the effects of kisspeptin/kisspeptin receptor signalling in the lung, focusing on airway-associated adipose tissue deposition and impact on airway structure–function. Wild-type, heterozygous and kisspeptin receptor knockout mice were studied at 6 or 8 weeks of age. Lung mechanics were assessed before and after methacholine challenge and were subsequently fixed for airway morphometry. A separate group of mice underwent glucose tolerance testing and bronchoalveolar lavage. At 6 weeks of age, kisspeptin/kisspeptin receptor signalling did not affect body adiposity, airway inflammation, wall structure or function. Despite no differences in body adiposity, there was a greater accumulation of airway-associated adipose tissue in knockout mice. By 8 weeks of age, female knockout mice displayed a non-diabetic phenotype with increased body adiposity but not males. Airway-associated adipose tissue area was also increased in both knockout females and males at 8 weeks of age, but again no other respiratory abnormality was apparent. In summary, airway-associated adipose tissue is decoupled from body adiposity in prepubescent mice which supports a genetic susceptibility to fatty deposits localised to the airway wall. There was no evidence that airway-associated adipose tissue drives pathology or respiratory impairment in the absence of other environmental exposures.

## Introduction

Patients with asthma experience symptoms including cough, wheeze and shortness of breath which are underpinned by airway inflammation, remodelling (e.g., thickening of the airway smooth muscle [ASM] layer [1]) and lung function impairment. Disease severity is increased in patients with asthma who are also obese, where excessive adipose tissue accumulation worsens symptoms and reduces responsiveness to controller therapy [2]. Our research group has identified and quantified adipose tissue within the airway wall, dubbed ‘airway-associated adipose tissue’, which was increased in overweight and obese patients, and positively correlated to airway wall thickness [3]. Using pig lungs, we also demonstrated that airway-associated adipose tissue releases pro-inflammatory adipokines [4]. Together these findings support a direct or indirect role of airway-associated adipose tissue in severe asthma patients who are also obese.

However, when closely examining the positive linear relationship between body mass index (BMI) and airway-associated adipose tissue [3], it is evident that BMI accounts for only ∼40% of fat accumulation in the airway wall, suggesting other contributing factors may be at play. In particular, there is a known genetic predisposition to both obesity [5] and asthma development [6]. A gene of interest in the lung is kisspeptin (*Kiss1*) and its receptor (*Kiss1r*), which have been shown to abate proliferation of ASM cells *in vitro* with reduced expression in ASM cells from asthma subjects compared with healthy subjects [7]. Activation of the *Kiss1*/*Kiss1r* signalling pathway also attenuates histopathological lung abnormalities present in pulmonary fibrosis by reducing inflammatory cell infiltration, α-smooth muscle actin production and collagen deposition [8]. Outside the lung, both *Kiss1* and *Kiss1r* are expressed in peripheral organs including the liver and white adipose tissue (WAT, [9]) and contribute to regulation of energy expenditure, adiposity and glucose metabolism [10]. Impaired *Kiss1*/*Kiss1r* signalling leads to spontaneous development of obesity in adult female mice, with greater body weight, higher abdominal adiposity, reduced glucose tolerance [11], diminished thermogenic capacity within brown adipose tissue and a lower core body temperature [9]. Effects are sex-dependent, mediated through *Kiss1* expression in the hypothalamus and modification of the downstream release of sex hormones [12].

The primary aim of the present study was to examine a possible genetic determinant of airway-associated adipose tissue. We used a *Kiss1r* knockout (KO) mouse model to quantify airway-associated adipose tissue and body adiposity at prepubescent and young adult timepoints, in both males and females. A secondary aim was to assess abnormalities in airway structure–function and inflammation in response to disruption of *Kiss1*/*Kiss1r* signalling.

## Methods

### Kiss1r KO mouse model

**Figure 1:**
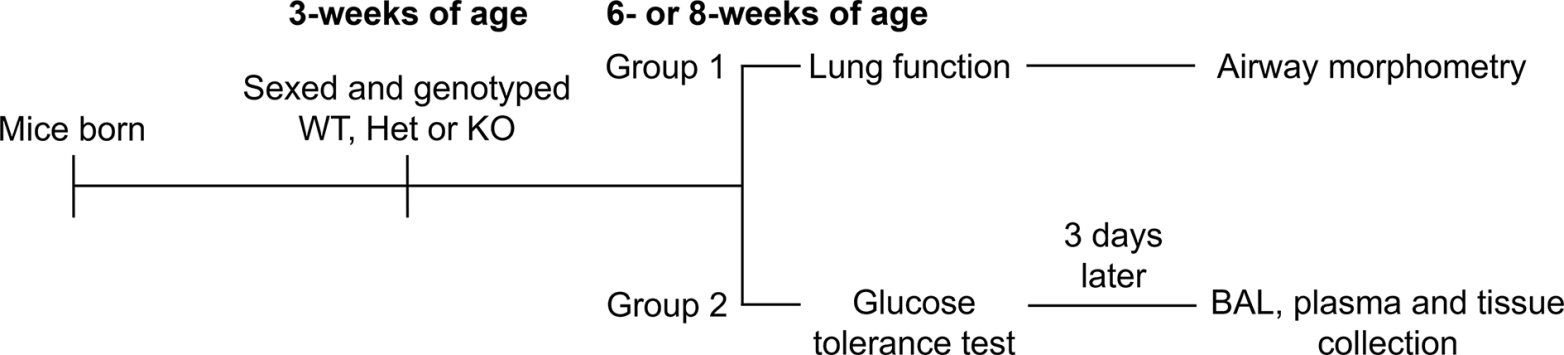
Mouse study protocol Male and female WT, Het and KO mice were randomly assigned into two groups for experimentation. Experiments were performed on mice at either 6 or 8 weeks of age. BAL, bronchoalveolar lavage; Het, heterozygous; KO, knockout; WT, wild-type.

Procedures were approved by The University of Western Australia Animal Ethics Committee (RA/3/100/1696) in accordance with National Health and Medical Research Council guidelines. Mice of *Kiss1r* wild-type (WT), heterozygous (Het) and KO genotypes were generated by mating Het breeders carrying the retroviral insertion in the *Kiss1r* gene on a C57BL/6J background (generations 19–25, [11]). Mice were originally generated by Omeros Corporation (Seattle, Washington, U.S.A.) and rederived at the Biomedical Research Facility, The University of Western Australia (Shenton Park, Western Australia, Australia). At 3 weeks of age, mice were genotyped by PCR after ear punch [13]. From birth, mice were housed at the Biomedical Research Facility, The University of Western Australia and after genotyping, mice were transferred to the Pre-Clinical Facility at The University of Western Australia (Crawley, Western Australia, Australia) for experiments. Littermates were housed on a 12:12 light:dark cycle and maintained at a temperature between 18 and 24°C. Mice had access to *ad libitum* water and standard rodent chow. Male and female genotypes (6 weeks of age ‘prepubescent’, 8 weeks of age ‘young adult’) were randomly assigned into two experimental groups (Figure 1). Sample sizes for each outcome are listed in the Results, Figure Legends or Tables. Body weight, snout-vent length and abdominal circumference were recorded during experimentation.

### Lung function

Group 1 mice were anaesthetised with an *i.p*. injection of ketamine (0.4 mg/g body weight) and xylazine (0.02 mg/g body weight), tracheostomised and subsequently ventilated at 250 breaths/min on a FlexiVent system (FX module 1, flexiWare version 7.5, SCIREQ). Lung volume history was standardised by three slow inflation-deflation manoeuvrers from 0 to 20 cmH_2_O transrespiratory pressure [14]. The change in lung volume from 0 to 20 cmH_2_O transrespiratory pressure was normalized to body weight. The forced oscillation technique was used to measure respiratory impedance before and after methacholine (MCh, acetyl-β-methacholine chloride; Merck KgaA) challenge (saline-30 mg/ml). Airway resistance (*R*_aw_), tissue damping (*G*) and tissue elastance (*H*) were derived from respiratory impedance. The delta change was calculated as the difference from saline to MCh at 30 mg/ml (i.e., Δ*R*_aw_, Δ*G* and Δ*H*).

### Airway morphometry

Following the final dose of MCh, a 0.1 ml *i.p*. injection of atropine (600 µg/ml) was administered, and 10 min later mice were euthanized with an overdose of ketamine and xylazine. After euthanasia (Group 1), lungs were inflation-fixed in 4% formaldehyde at 10 cmH_2_O transpulmonary pressure. Transverse sections (5 µm) from three zones within the left lung (upper, middle and lower regions) were stained with hematoxylin and eosin (H&E, [15]) to quantify airway size (perimeter of basement membrane, *P*_bm_) and areas of airway-associated adipose tissue, epithelium, ASM, inner (WA_i_), outer (WA_o_) and total wall (WA_t_) using Stereo Investigator Software (version 10.42.1, MBF Bioscience). Identification of airway-associated adipose tissue has been previously validated [4] where in H&E-stained sections, clustered adipocytes were generally spherical with a single nucleus localised to the cell membrane, adopting a signet ring appearance [3] typical of WAT morphology. Normalization to airway size was achieved by square rooting area and division by *P*_bm_. Case means were obtained for central and peripheral airways by calculating the average over the three measured zones for each mouse.

### Glucose tolerance

Group 2 mice were re-housed in clean cages to void food remnants with access to water *ad libitum* and fasted for 5 h prior to *i.p*. glucose injection (1 g/kg body weight). Blood sampled from the tail-tip was used to measure blood glucose levels at baseline and 15, 30, 45, 60, 90 and 120 min post-glucose injection using a glucometer (Accu-Chek Performa, Roche; [16]). Assessment of glucose tolerance was determined by calculating area under the curve for blood glucose levels over 2 h.

### Bronchoalveolar lavage, tissue collection and analyses

Three days following glucose tolerance testing (Group 2), mice were euthanized by an overdose *i.p*. injection of ketamine and xylazine and then tracheostomised for bronchoalveolar lavage (BAL) fluid cell counts [17]. The BAL fluid was centrifuged at 400 ***g*** for 4 min and the cell pellet was resuspended in phosphate-buffered solution. Lavage samples were processed for total BAL cell counts as previously described [18]; resuspended cells were identified using trypan blue staining and total cell counts were performed using a haemocytometer. Lungs and WAT (female, ovarian region; male, epididymal region) were dissected and weighed. Right lungs from mice at 6 weeks of age were snap frozen to analyse the expression of *Kiss1*. Homogenised lung tissue was treated with DNase (RQ1 RNase-free DNase, Promega) during RNA isolation to avoid genomic DNA contamination. Samples were analysed by quantitative real-time PCR (qPCR) using designer primers (Table 1) with QuantiNova SYBR Green PCR Master Mix (Qiagen), using CFX384 Touch Real-Time PCR Detection System (Bio-Rad). Standard curves were used to interpolate *C*t values of target mRNA and normalised to reference genes: peptidylprolyl isomerase A (PPIA), succinate dehydrogenase (SDHA) and TATA-binding protein (TBP).

**Table 1:** Designer primer sequences for mouse *Kiss1*, *Kiss1r* and reference genes from Geneworks

Gene	Product size (bp)	Forward sequence	Reverse sequence
*Kiss1*	126	5′-CTCTGTGTCGCCACCTATGG-3′	5′-AGGCTTGCTCTCTGCATACC-3′
*Kiss1r*	172	5′-CTGTCAGCCTCAGCATCTGG-3′	5′-AGCAGCGGCAGCAGATATAG-3′
PPIA	127	5′-AGCATACAGGTCCTGGCATC-3′	5′-TTCACCTTCCCAAAGACCAC-3′
SDHA	149	5′-TGGGGAGTGCCGTGGTGTCA-3′	5′-CTGTGCCGTCCCCTGTGCTG-3′
TBP	113	5′-GGGAGAATCATGGACCAGAA-3′	5′-CCGTAAGGCATCATTGGACT-3′

*Kiss1*, kisspeptin; *Kiss1r*, kisspeptin receptor; PPIA, peptidylprolyl isomerase; SDHA, succinate hydrogenase; TBP, TATA box binding protein.

### Metabolic assay

Cardiac puncture (post-mortem) was also performed on mice at 6 weeks of age from Group 2. Blood samples were centrifuged at 2000 ***g*** for 5 min at 4°C. The Milliplex™ Mouse Metabolic Magnetic Bead Panel Assay (Cat# MMHE-44K, Merck KGaA) was used to measure plasma biomarker concentrations: C-peptide, gastric inhibitory peptide, glucagon, insulin, leptin, peptide YY and resistin. Analyte fluorescence was analysed with 5PL logarithmic regression.

### Statistical analysis

Genotype, sex or age effects were analysed by two- or three-way ANOVA. Data were transformed to satisfy assumptions of normality and homoscedasticity as required with SigmaPlot (version 14.5, Systat Software Inc.). Non-parametric data were compared between genotypes using a Kruskal–Wallis test, and between sex with a Mann–Whitney *U* test. Functional outcomes (*R*_aw_,* G* and *H*) were compared between genotype and sex, before and after MCh challenge. Graphical analyses were conducted using PRISM (version 9.5.1, GraphPad). Data are presented as mean ± SEM with *P*<0.05 considered statistically significant.

## Results

### Kiss1 and Kiss1r expression in the lung

**Figure 2:**
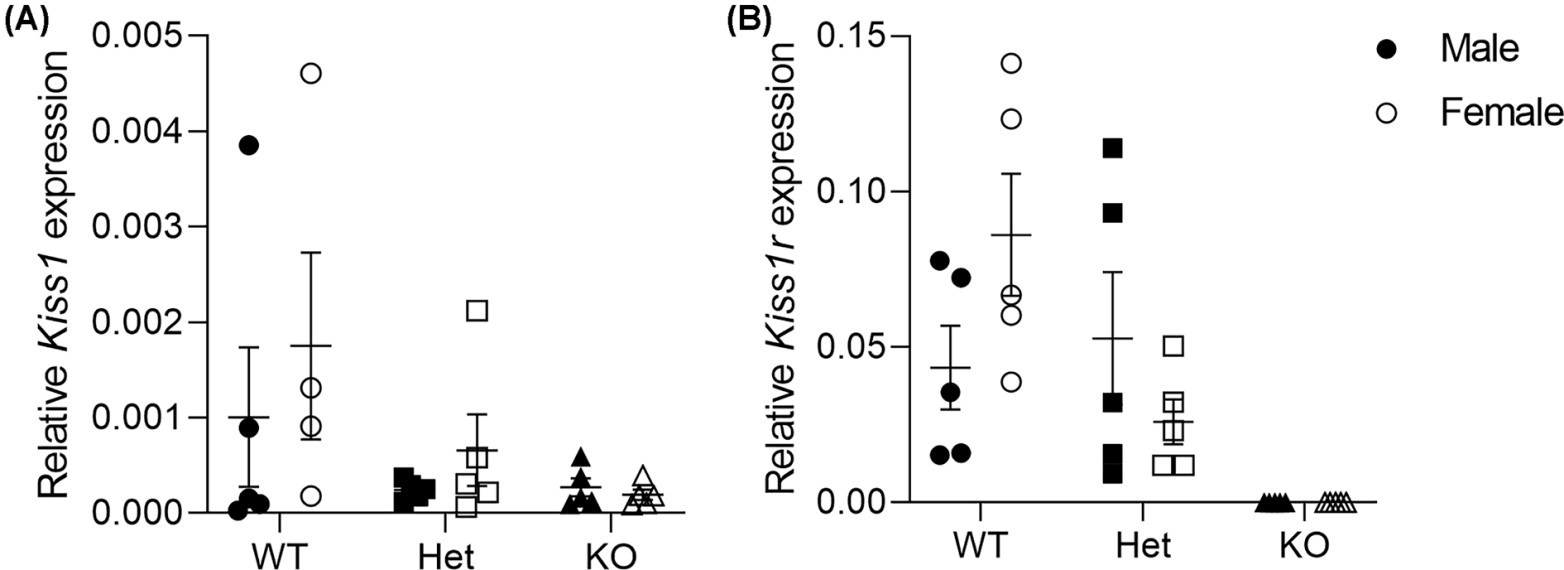
Relative expression of *Kiss1* and *Kiss1r* genes in mouse lungs at 6 weeks of age The gene expression for *Kiss1* (**A**) and *Kiss1r* (**B**) were normalised to reference genes PPIA, SDHA and TBP. Data are presented as mean ± SEM, where closed circles represent WT males, open circles represent WT females, closed squares represent Het males, open squares represent Het females, closed triangles represent KO males, open triangles represent KO females. *Kiss1*; WT, *n* = 5 males, *n* = 4 females; Het, *n* = 5 males, *n* = 5 females; KO, *n* = 5 males, *n* = 5 females and *Kiss1r*; WT, *n* = 5 males, *n* = 5 females; Het, *n* = 5 males, *n* = 5 females; KO, *n* = 5 males, *n* = 5 females. Het, Heterozygous; *Kiss1*, kisspeptin; *Kiss1r*, kisspeptin receptor; KO, knockout; PPIA, peptidylprolyl isomerase A; SDHA, succinate dehydrogenase; TBP, TATA-binding protein; WT, wild-type.

The *Kiss1* gene was expressed in the mouse lung of all groups (genotype, *P*=0.193; sex, *P*=0.261; Figure 2A). Expression of the *Kiss1r* gene was similar between WT and Het groups (genotype, *P*=0.141; sex, *P*=0.635) and absent in KO mice (Figure 2B).

### Anthropometrics—body size, adiposity and tissue weights

**Figure 3:**
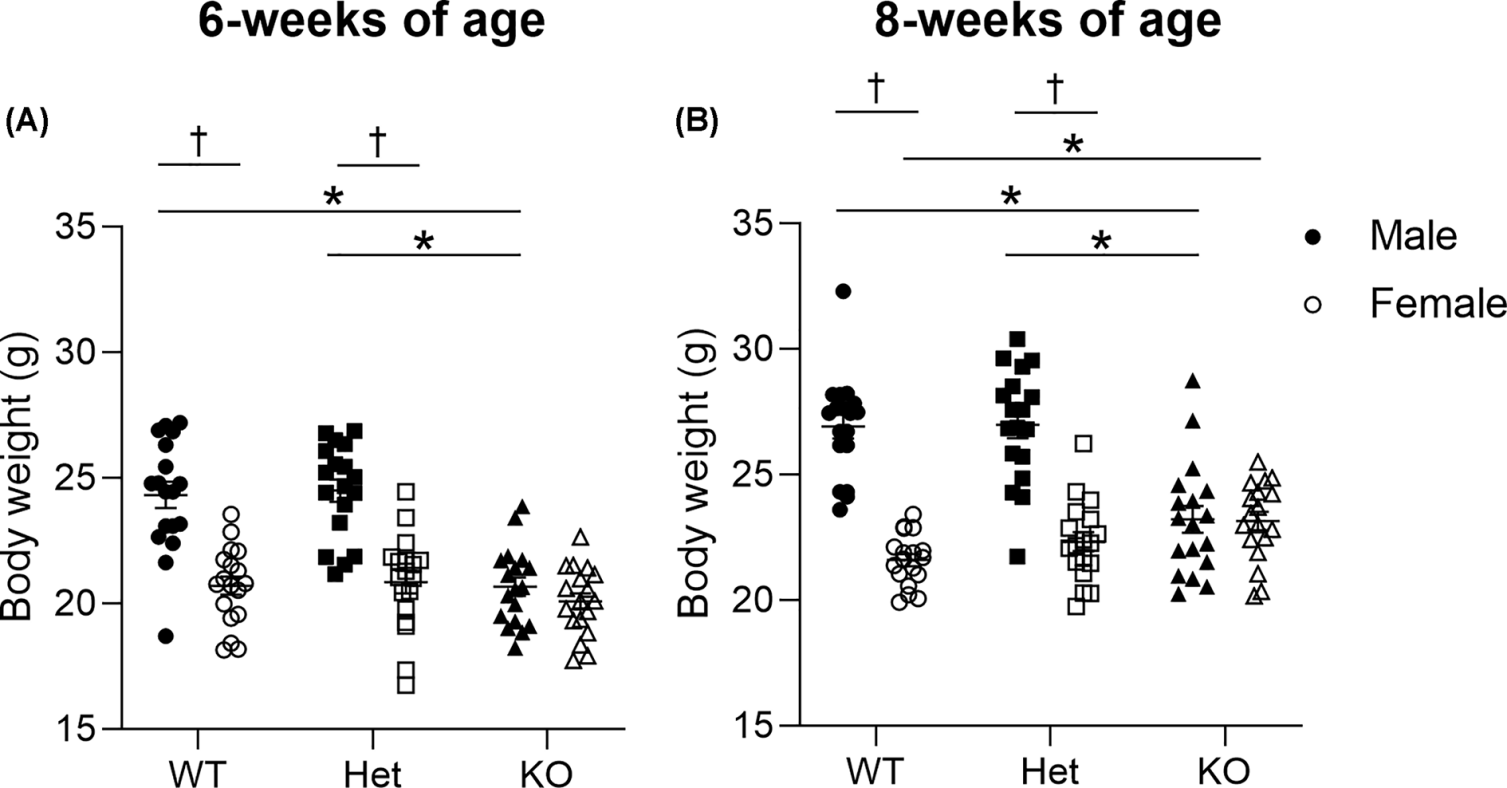
Mouse body weight Six weeks (**A**) and eight weeks (**B**) of age. * denotes genotype effect and † denotes significant sex effect (*P*<0.05). Data are presented as mean ± SEM. Closed circles represent WT males, open circles represent WT females, closed squares represent Het males, open squares represent Het females, closed triangles represent KO males, open triangles represent KO females. Six and eight weeks of age: WT, *n* = 18 males, *n* = 18 females; Het, *n* = 18 males, *n* = 18 females; KO, *n* = 18 males, *n* = 18 females; Het, Heterozygous; KO, knockout; WT, wild-type.

At 6 weeks of age, WT and Het males were greater in body weight than KO males (*P*<0.001, Figure 3A). Snout-vent length was also larger in Het males than KO males (*P*=0.045, Table 2). Body weight of female mice was comparable between genotypes (*P*>0.05, Figure 3A). There was no effect of genotype on absolute WAT (*P*=0.078) or WAT normalised to body weight (*P*=0.778), and therefore no evidence of increased body adiposity in prepubescent animals (Table 3). Similarly, there were no genotypic differences in absolute (*P*=0.198) or relative (*P*=0.126) lung weights (Table 3).

**Table 2:** Mouse body characteristics

	Six weeks of age						Eight weeks of age					
	WT		Het		KO		WT		Het		KO	
Measurement	Male (*n*=18)	Female (*n*=18)	Male (*n*=18)	Female (*n*=18)	Male (*n*=18)	Female (*n*=18)	Male (*n*=18)	Female (*n*=18)	Male (*n*=18)	Female (*n*=18)	Male (*n*=18)	Female (*n*=18)
**Snout-vent length (cm)**	9.6 ± 0.1*†‡	9.3 ± 0.1	9.8 ± 0.1*†	9.1 ± 0.1	9.3 ± 0.1	9.2 ± 0.2	9.9 ± 0.1*†	9.6 ± 0.1	10.1 ± 0.1*†	9.7 ± 0.1	9.7 ± 0.1	9.8 ± 0.1
**Abdominal circumference (cm)**	6.9 ± 0.1^†^	6.4 ± 0.1	6.8 ± 0.1^†^	6.4 ± 0.1	6.4 ± 0.1^†^	6.3 ± 0.1	6.5 ± 0.1*†	5.9 ± 0.1	6.5 ± 0.1*	6.2 ± 0.1	5.9 ± 0.1	6.0 ± 0.1

* denotes *P*<0.05 compared with KO, ^‡^ denotes *P*<0.05 compared with Het and † denotes *P*<0.05 compared with females. Data are presented as mean ± SEM. Het, heterogenous; KO, knockout; WT, wild-type.

**Table 3:** Tissue weights from mice. Total WAT and lung weights were obtained post-mortem and normalised to body weight

	Six weeks of age						Eight weeks of age					
	WT		Het		KO		WT		Het		KO	
Measurement	Male (*n*=8)	Female (*n*=8)	Male (*n*=8)	Female (*n*=8)	Male (*n*=8)	Female (*n*=8)	Male (*n*=8)	Female (*n*=8)	Male (*n*=8)	Female (*n*=8)	Male (*n*=8)	Female (*n*=8)
**WAT weight (g)**	0.231 ± 0.026†	0.188 ± 0.013	0.282 ± 0.016†	0.176 ± 0.007	0.195 ± 0.015†	0.175 ± 0.018	0.305 ± 0.017†	0.221 ± 0.012*	0.342 ± 0.024†	0.241 ± 0.024*	0.292 ± 0.018†	0.401 ± 0.017
**WAT weight (% of body weight)**	0.947 ± 0.082†	0.892 ± 0.044	1.088 ± 0.058†	0.832 ± 0.035	0.944 ± 0.064†	0.881 ± 0.084	1.169 ± 0.051	1.016 ± 0.050*	1.238 ± 0.083	1.063 ± 0.100*	1.299 ± 0.071†	1.722 ± 0.071
**Total lung weight (g)**	0.282 ± 0.009	0.276 ± 0.012	0.290 ± 0.005	0.261 ± 0.010	0.252 ± 0.013	0.256 ± 0.012	0.324 ± 0.016	0.271 ± 0.012	0.318 ± 0.011	0.289 ± 0.010	0.288 ± 0.013	0.298 ± 0.016
**Total lung weight (% of body weight)**	1.182 ± 0.033†	1.326 ± 0.070	1.121 ± 0.018†	1.231 ± 0.035	1.274 ± 0.050†	1.297 ± 0.061	1.257 ± 0.080	1.247 ± 0.057	1.154 ± 0.038	1.284 ± 0.029	1.282 ± 0.047	1.282 ± 0.066

* denotes *P*<0.05 compared with KO and † denotes *P*<0.05 compared with females. Data are presented as mean ± SEM. Het, heterogenous; KO, knockout; WAT, white adipose tissue; WT, wild-type.

At 8 weeks of age, female KO mice had a greater body weight than female WT mice (*P*=0.039, Figure 3B). Despite no differences in female snout-vent length and abdominal circumference between genotypes (*P*>0.05), female KO had a greater absolute WAT and relative WAT weights (*P*<0.001, Table 3) than both female WT and Het mice, i.e., KO female mice had developed increased body adiposity. Within males, WT and Het mice were greater in body weight and abdominal circumference than KO mice (*P*<0.001, Figure 3B), with a greater snout-vent length in Het compared with KO mice (*P*<0.001, Table 2). However, there were no genotypic differences in absolute or relative WAT weights between males (*P*>0.05, Table 3). Both absolute (*P*=0.001) and relative (*P*<0.001) WAT weights were greater in female KO than male KO mice. Absolute (genotype, *P*=0.727; sex, *P*=0.052) and relative (genotype, *P*=0.605; sex, *P*=0.439) lung weights were comparable between mice (Table 3).

Compared with female counterparts, at 6 weeks of age WT and Het males had a greater body weight (*P*<0.001, Figure 3A). Generally, males had a greater abdominal circumference (*P*=0.001, Table 2), absolute WAT (*P*<0.001) and relative WAT weights (*P*=0.040) than females (Table 3). While absolute lung weight was comparable between sexes (*P*=0.158), females had a greater relative lung weight than males (*P*=0.043, Table 3). When looking at differences between sexes at 8 weeks of age, compared with WT and Het females, male counterparts had a greater body weight (*P*<0.001, Figure 3B) and snout-vent length (*P*<0.05, Table 2) with a greater abdominal circumference in male WT than female WT (*P*<0.001, Table 2).

### Lung function

**Figure 4:**
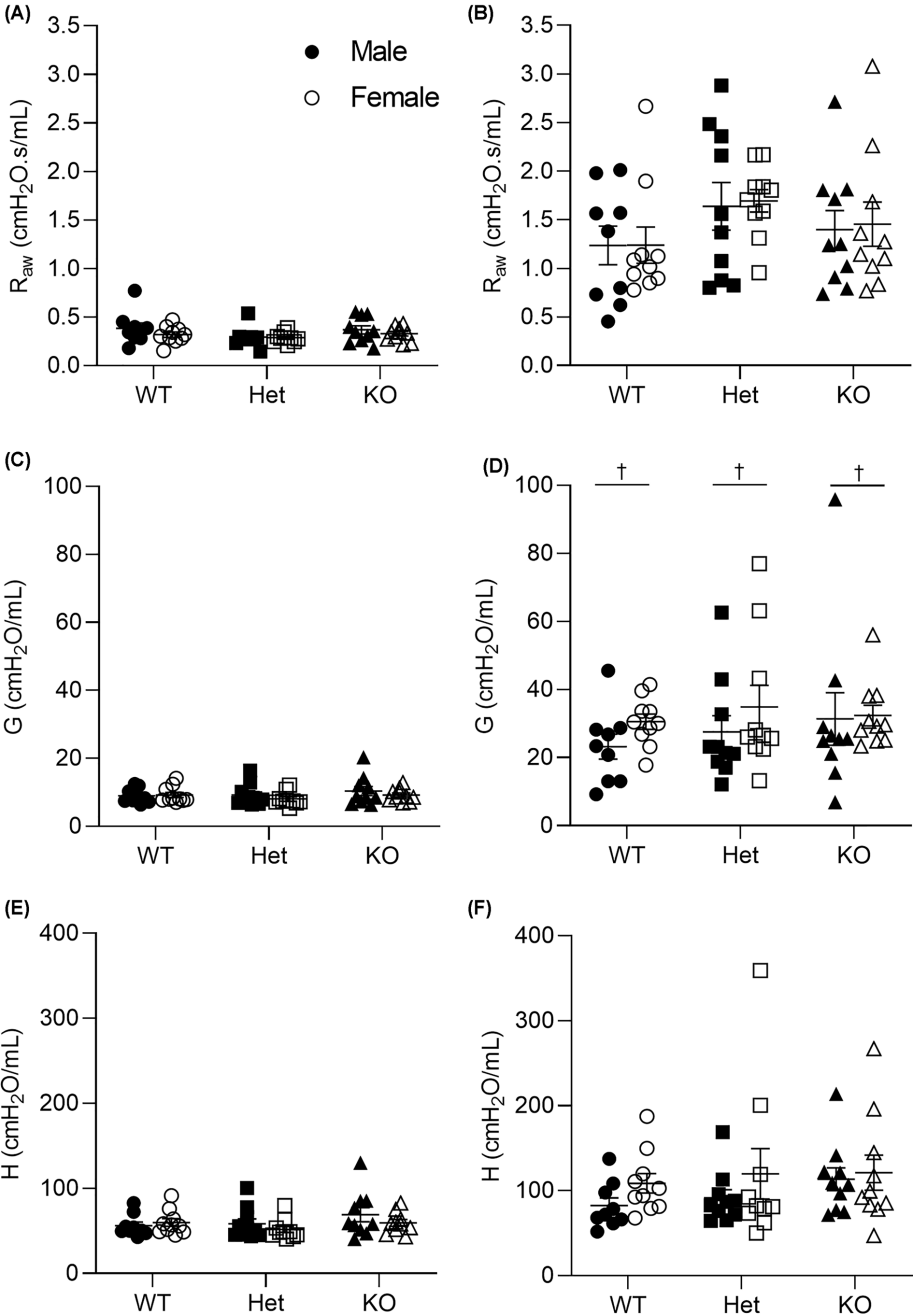
Lung function at 6 weeks of age Airway resistance before (**A**) and after MCh (30 mg/ml MCh, (**B**)). Airway tissue damping before (**C**) and after MCh (**D**). Airway tissue elastance before (**E**) and after MCh (**F**). † denotes significant sex effect (*P*<0.05) and data are presented as mean ± SEM. Closed circles represent WT males, open circles represent WT females, closed squares represent Het males, open squares represent Het females, closed triangles represent KO males, open triangles represent KO females. WT, *n* = 9 males, *n* = 10 females; Het, *n* = 10 males, *n* = 10 females; KO, *n* = 10 males, *n* = 10 females. *G*, airway tissue damping; *H*, airway tissue elastance; Het, heterozygous; KO, knockout; *R*_aw_, airway resistance; WT, wild-type.

**Figure 5:**
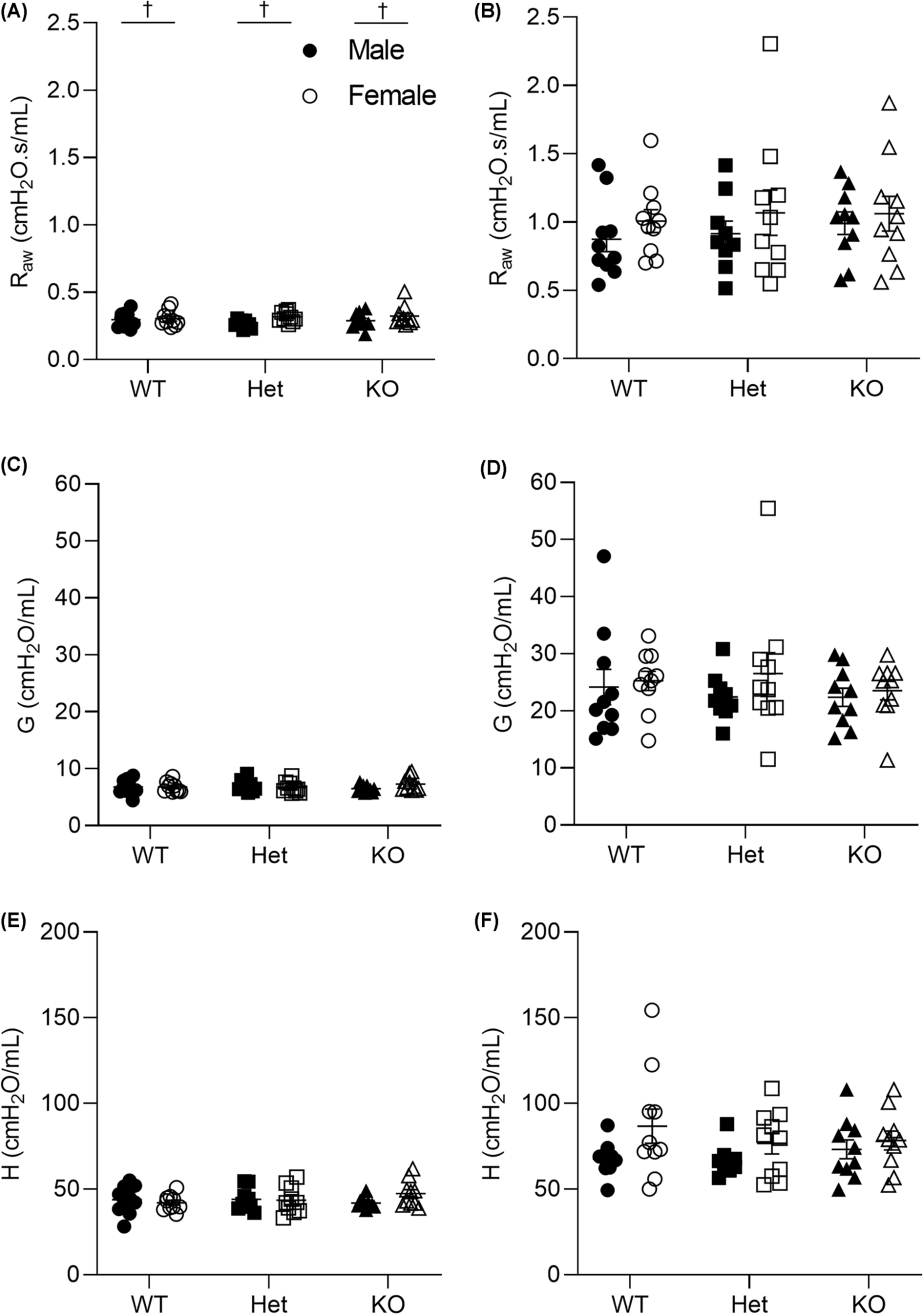
Lung function at 8 weeks of age Airway resistance before (**A**) and after MCh (30 mg/ml MCh; (**B**)). Airway tissue damping before (**C**) and after MCh (**D**). Airway tissue elastance before (**E**) and after MCh (**F**). † denotes significant sex effect (*P*<0.05), and data are presented as mean ± SEM. Closed circles represent WT males, open circles represent WT females, closed squares represent Het males, open squares represent Het females, closed triangles represent KO males, open triangles represent KO females. WT, *n* = 10 males; *n* = 10 females; Het, *n* = 9 males, *n* = 10 females; KO, *n* = 10 males, *n* = 10 females. *G*, airway tissue damping; *H*, airway tissue elastance; Het, heterozygous; KO, knockout; *R*_aw_, airway resistance; WT, wild-type.

At 6 weeks of age, *R*_aw_ (Figure 4A,B), *G* (Figure 4C,D) and *H* (Figure 4E,F) before and after MCh challenge were comparable between genotypes (*P*>0.05). There were no genotypic differences in Δ*R*_aw_ (*P*=0.051), Δ*G* (*P*=0.676) or Δ*H* (*P*=0.263) in response to MCh challenge. Similarly, at 8 weeks of age, there were no genotypic differences in *R*_aw_ (Figure 5A,B), *G* (Figure 5C,D) or *H* (Figure 5E,F) before or after MCh challenge (*P*>0.05), nor were there genotypic differences in Δ*R*_aw_ (*P*=0.779), Δ*G* (*P*=0.950) or Δ*H* (*P*=0.579) to MCh. Sex differences at 6 weeks of age showed that female mice had greater G after MCh than males (*P*=0.040, Figure 4D), but this was not observed in *R*_aw_ (*P*=0.569, Figure 4B) or *H* (*P*=0.175, Figure 4F) after MCh. Females had a greater Δ*G* (*P*=0.007) and Δ*H* (*P*=0.017) than males, but both sexes had a similar Δ*R*_aw_ (*P*=0.415). By 8 weeks of age, females had a higher *R*_aw_ before MCh (*P*=0.020, Figure 5A) and Δ*H* (*P*=0.034) than males. There were no differences in Δ*R*_aw_ (*P*=0.463) and Δ*G* (*P*=0.128) between males and females.

Females had a greater Δ lung volume relative to body weight than males but there were no differences between genotypes (WT males [*n*=10], 0.018 ± 0.001 ml/g; WT females [*n*=10], 0.020 ± 0.001 ml/g; Het males [*n*=10], 0.017 ± 0.002 ml/g; Het females [*n*=10], 0.022 ± 0.001 ml/g; KO males [*n*=10], 0.018 ± 0.002 ml/g; KO females [*n*=10], 0.020 ± 0.001 ml/g; genotype, *P*=0.859; sex, *P*=0.006). The relative Δ lung volume was greater in KO males compared with WT and Het males (*P*=0.009), which is likely driven by the reduced body weight of KO males. Female WT and Het had a greater relative Δ lung volume than male counterparts (*P*<0.05; WT males, 0.018 ± 0.001 ml/g; WT females, 0.023 ± 0.001 ml/g; Het males, 0.019 ± 0.002 ml/g; Het females, 0.024 ± 0.001 ml/g; KO males, 0.024 ± 0.001 ml/g; KO females, 0.021 ± 0.001 ml/g).

### Airway-associated adipose tissue

**Figure 6:**
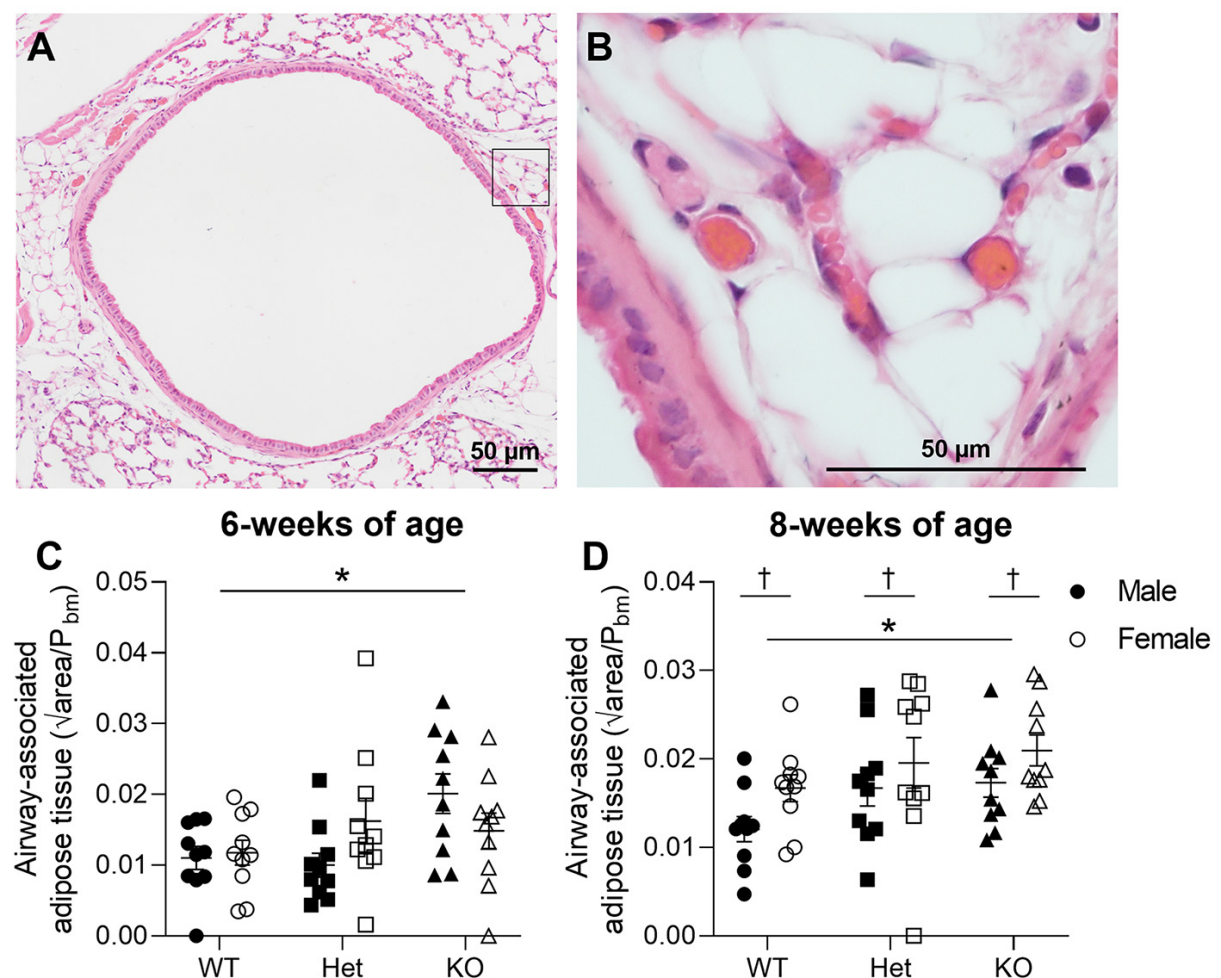
Airway-associated adipose tissue Histological image of H&E-stained central airway in KO mouse at ×10 magnification (**A**). Black box delineates magnified area of airway-associated adipose tissue at ×40 magnification (**B**). Quantified airway-associated adipose tissue in mice at 6 weeks (**C**) and 8 weeks (**D**) of age. * denotes significant genotype effect and † denotes significant sex effect (*P*<0.05). Data are presented as mean ± SEM and scale bar represents 50 µm. Closed circles represent WT males, open circles represent WT females, closed squares represent Het males, open squares represent Het females, closed triangles represent KO males, open triangles represent KO females. Six and eight weeks of age: WT, *n* = 10 males, *n* = 10 females; Het, *n* = 10 males, *n* = 10 females; KO, *n* = 10 males; *n* = 10 females. H&E, hematoxylin and eosin; Het, heterozygous; KO, knockout; WT, wild-type.

Airway-associated adipose tissue was typically observed in central airways (∼96%) and was less prevalent in peripheral airways (∼32%) at 6 weeks of age. Unlike body adiposity, KO mice at 6 weeks of age had ∼53% more airway-associated adipose tissue (Figure 6A,B) compared with WT mice (*P*=0.034) but contained a similar amount to Het mice (*P*>0.05, Figure 6C). There was no independent effect of sex on airway-associated adipose tissue (*P*=0.770).

At 8 weeks of age, airway-associated adipose tissue was present in most centrally located airways (∼98%) but also became more prevalent in peripheral airways (∼62%). Again, KO mice had a greater accumulation of airway-associated adipose tissue in central airways than WT mice (*P*=0.026, Figure 6D), but not compared with Het mice (*P*=0.471). Females had a greater airway adiposity than males (*P*=0.013, Figure 6D). When compared with mice at 6 weeks of age, mice at 8 weeks of age had greater airway-associated adipose tissue in central airways, which was consistently increased in KO than WT mice (age, *P*=0.013; genotype, *P*=0.002; sex, *P*=0.242). Given that over a third of peripheral airways did not contain airway-associated adipose tissue, statistical analyses could not be conducted at 6 or 8 weeks of age.

### Airway wall structure

At 6 weeks of age, WT mice had greater WA_o_ in peripheral airways than KO mice (*P*=0.032), but there was no effect of genotype on other airway wall structures (*P*>0.05, Table 5). By 8 weeks of age, ASM layer thickness was greater in peripheral airways of female KO compared with female Het mice (*P*=0.005, Table 5), but this was not observed in central airways (*P*=0.125). At 8 weeks of age, there was also an increase in WA_i_ of peripheral airways in KO mice compared with WT mice (*P*=0.026).

When looking at the effect of sex at 6 weeks of age, males had a greater ASM layer thickness than females in central (*P*=0.022) and peripheral airways (*P*=0.005), driving differences observed in WA_i_ (*P*<0.05, Table 4). Epithelial area in central airways was greater in males than females (*P*=0.048), but there were no sex differences in *P*_bm_ (*P*=0.175), WA_o_ (*P*=0.675) or WA_t_ (*P*=0.375). In peripheral airways, WA_o_ was greater in males compared with females (*P*=0.040, Table 4). Males also had a greater WA_t_ (*P*=0.003) than females, with no sex differences in *P*_bm_ (*P*=0.111) or epithelial area (*P*=0.124) in peripheral airways. By 8 weeks of age, females had a greater WA_o_ in central airways than males (*P*=0.043, Table 5). Males had a greater epithelial area in central airways (*P*=0.048) and WA_t_ in peripheral airways (*P*=0.003) than females (Table 5). There were no sex differences in central airway *P*_bm_ (*P*=0.563), epithelial area (*P*=0.789), ASM (*P*=0.105), WA_i_ (*P*=0.096) or WA_t_ (*P*=0.529, Table 5). Again, the peripheral airways of mice at 8 weeks of age were comparable between sex for structural measurements of *P*_bm_ (*P*=0.669), epithelial area (*P*=0.752), WA_o_ (*P*=0.287) and WA_t_ (*P*=0.602, Table 5).

**Table 4:** Quantification of airway wall structures at 6 weeks of age

	Central airways						Peripheral airways					
	WT		Het		KO		WT		Het		KO	
Structure	Male (*n*=10)	Female (*n*=10)	Male (*n*=10)	Female (*n*=10)	Male (*n*=10)	Female (*n*=10)	Male (*n*=10)	Female (*n*=10)	Male (*n*=10)	Female (*n*=10)	Male (*n*=10)	Female (*n*=10)
***P*_bm_ (mm)**	1.727 ± 0.088	1.925 ± 0.108	1.740 ± 0.074	1.740 ± 0.122	1.664 ± 0.102	1.790 ± 0.066	0.762 ± 0.055	0.859 ± 0.096	0.714 ± 0.076	0.749 ± 0.079	0.588 ± 0.026	0.764 ± 0.072
**Epithelium (√area/*P*_bm_)**	0.100 ± 0.007†	0.088 ± 0.005	0.092 ± 0.003†	0.091 ± 0.005	0.096 ± 0.004†	0.090 ± 0.002	0.133 ± 0.006	0.128 ± 0.005	0.138 ± 0.006	0.135 ± 0.006	0.145 ± 0.003	0.133 ± 0.004
**ASM (√area/*P*_bm_)**	0.068 ± 0.004†	0.061 ± 0.002	0.068 ± 0.003†	0.066 ± 0.003	0.071 ± 0.003†	0.063 ± 0.002	0.079 ± 0.005†	0.065 ± 0.003	0.079 ± 0.005†	0.079 ± 0.004	0.079 ± 0.005†	0.067 ± 0.002
**WA_i_ (√area/*P*_bm_)**	0.072 ± 0.004†	0.065 ± 0.002	0.072 ± 0.003†	0.070 ± 0.004	0.075 ± 0.003†	0.068 ± 0.002	0.082 ± 0.005†	0.069 ± 0.002	0.084 ± 0.005†	0.080 ± 0.004	0.084 ± 0.005†	0.073 ± 0.002
**WA_o_ (√area/*P*_bm_)**	0.075 ± 0.005	0.073 ± 0.004	0.069 ± 0.003	0.077 ± 0.004	0.080 ± 0.003	0.078 ± 0.004	0.085 ± 0.004*†	0.080 ± 0.003*	0.086 ± 0.003†	0.086 ± 0.004	0.097 ± 0.003†	0.085 ± 0.002
**WA_t_ (√area/*P*_bm_)**	0.106 ± 0.005	0.098 ± 0.004	0.100 ± 0.003	0.105 ± 0.005	0.111 ± 0.003	0.104 ± 0.003	0.119 ± 0.005†	0.106 ± 0.003	0.120 ± 0.005†	0.119 ± 0.003	0.129 ± 0.005†	0.113 ± 0.002

* denotes *P*<0.05 compared with KO and † denotes *P*<0.05 compared with females. Data are presented as mean ± SEM. ASM, airway smooth muscle; Het, heterozygous; KO, knockout; *P*_bm_, perimeter of the basement membrane; WA_i_, inner wall area; WA_o_, outer wall area; WA_t_, total wall area; WT, wild-type.

**Table 5:** Quantification of airway wall structures at 8 weeks of age

	Central airways						Peripheral airways					
	WT		Het		KO		WT		Het		KO	
Structure	Male (*n*=10)	Female (*n*=10)	Male (*n*=10)	Female (*n*=10)	Male (*n*=10)	Female (*n*=10)	Male (*n*=10)	Female (*n*=10)	Male (*n*=10)	Female (*n*=10)	Male (*n*=10)	Female (*n*=10)
***P*_bm_ (mm)**	1.847 ± 0.098	1.946 ± 0.048	1.961 ± 0.089	1.879 ± 0.076	1.896 ± 0.064	1.939 ± 0.060	0.788 ± 0.076	0.811 ± 0.085	0.862 ± 0.097	0.852 ± 0.069	0.793 ± 0.051	0.850 ± 0.076
**Epithelium (√area/*P*_bm_)**	0.095 ± 0.005	0.088 ± 0.005	0.095 ± 0003	0.098 ± 0.005	0.089 ± 0.002	0.090 ± 0.003	0.138 ± 0.004	0.133 ± 0.005	0.133 ± 0.007	0.131 ± 0.006	0.132 ± 0.004	0.134 ± 0.008
**ASM (√area/*P*_bm_)**	0.065 ± 0.001	0.061 ± 0.001	0.065 ± 0.002	0.063 ± 0.002	0.067 ± 0.001	0.066 ± 0.002	0.067 ± 0.002	0.069 ± 0.003	0.072 ± 0.003†	0.063 ± 0.002*	0.074 ± 0.002	0.075 ± 0.003
**WA_i_ (√area/*P*_bm_)**	0.071 ± 0.002	0.066 ± 0.001	0.070 ± 0.002	0.068 ± 0.002	0.071 ± 0.001	0.070 ± 0.002	0.072 ± 0.002*	0.074 ± 0.003*	0.078 ± 0.003	0.071 ± 0.003	0.080 ± 0.002	0.081 ± 0.003
**WA_o_ (√area/*P*_bm_)**	0.080 ± 0.003†	0.081 ± 0.002	0.079 ± 0.002†	0.088 ± 0.002	0.080 ± 0.003†	0.081 ± 0.002	0.082 ± 0.003	0.082 ± 0.003	0.081 ± 0.004	0.083 ± 0.004	0.079 ± 0.003	0.085 ± 0.003
**WA_t_ (√area/*P*_bm_)**	0.107 ± 0.003	0.105 ± 0.002	0.105 ± 0.003	0.111 ± 0.003	0.108 ± 0.002	0.108 ± 0.002	0.109 ± 0.003	0.111 ± 0.004	0.113 ± 0.004	0.110 ± 0.003	0.113 ± 0.002	0.117 ± 0.004

* denotes *P*<0.05 compared with KO and † denotes *P*<0.05 compared with females. Data are presented as mean ± SEM. ASM, airway smooth muscle; Het, heterozygous; KO, knockout; *P*_bm_, perimeter of the basement membrane; WA_i_, inner wall area; WA_o_, outer wall area; WA_t_, total wall area; WT, wild-type.

### Glucose tolerance

**Figure 7:**
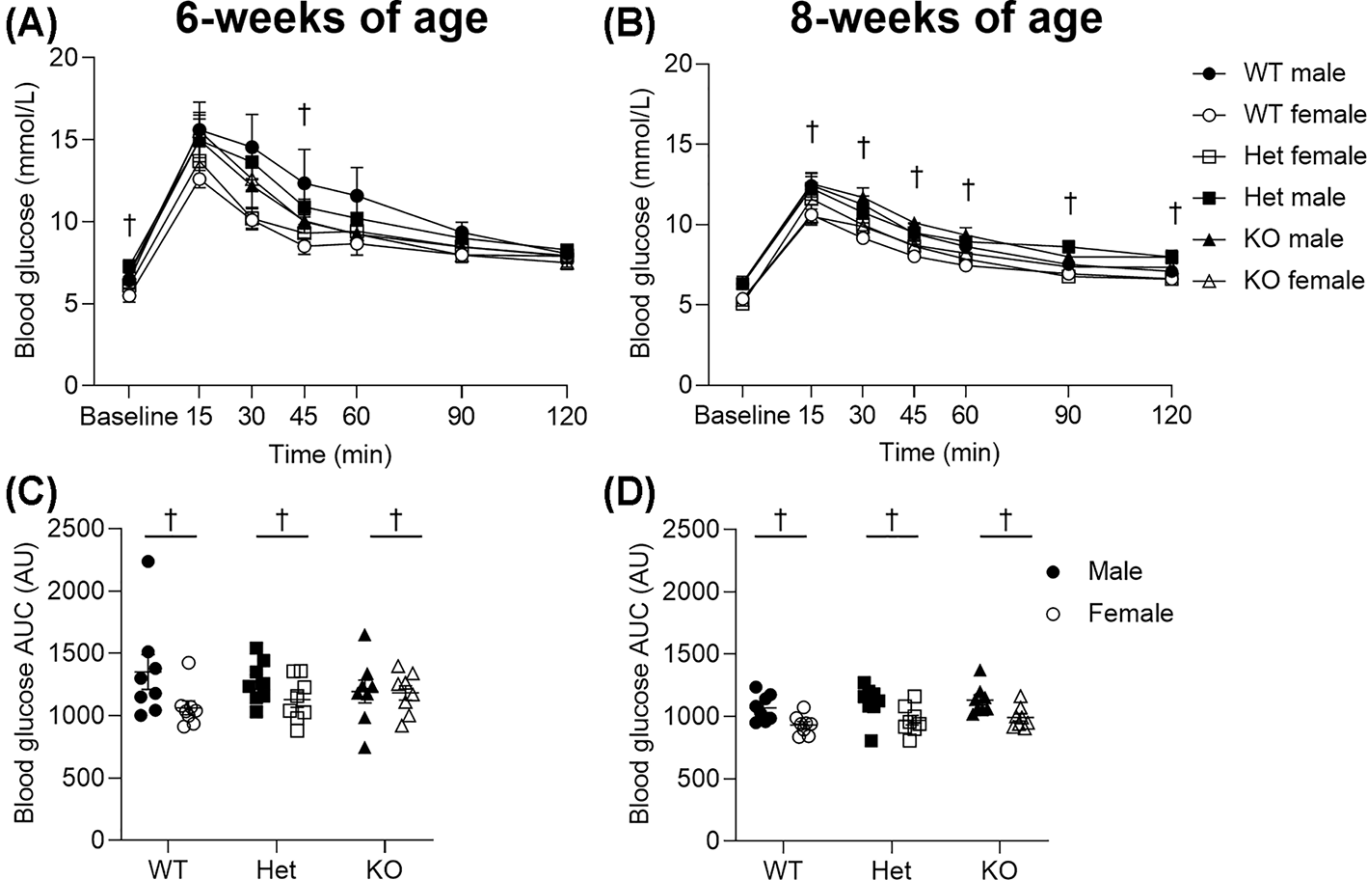
Blood glucose before and after glucose tolerance testing Changes in blood glucose over 2 h following bolus *i.p*. dose of glucose at 6 weeks (**A**) and 8 weeks (**B**) of age. The AUC for blood glucose at 6 weeks (**C**) and 8 weeks (**D**) of age. † denotes significant sex effect (*P*<0.05) and data are presented as mean ± SEM. Closed circles represent WT males, open circles represent WT females, closed squares represent Het males, open squares represent Het females, closed triangles represent KO males, open triangles represent KO females. Six and eight weeks of age: WT, *n* = 8 males, *n* = 8 females; Het, *n* = 8 males, *n* = 8 females; KO, *n* = 8 males, *n* = 8 females. AUC, area under the curve; Het, Heterozygous; KO, knockout; WT, wild-type.

**Figure 8:**
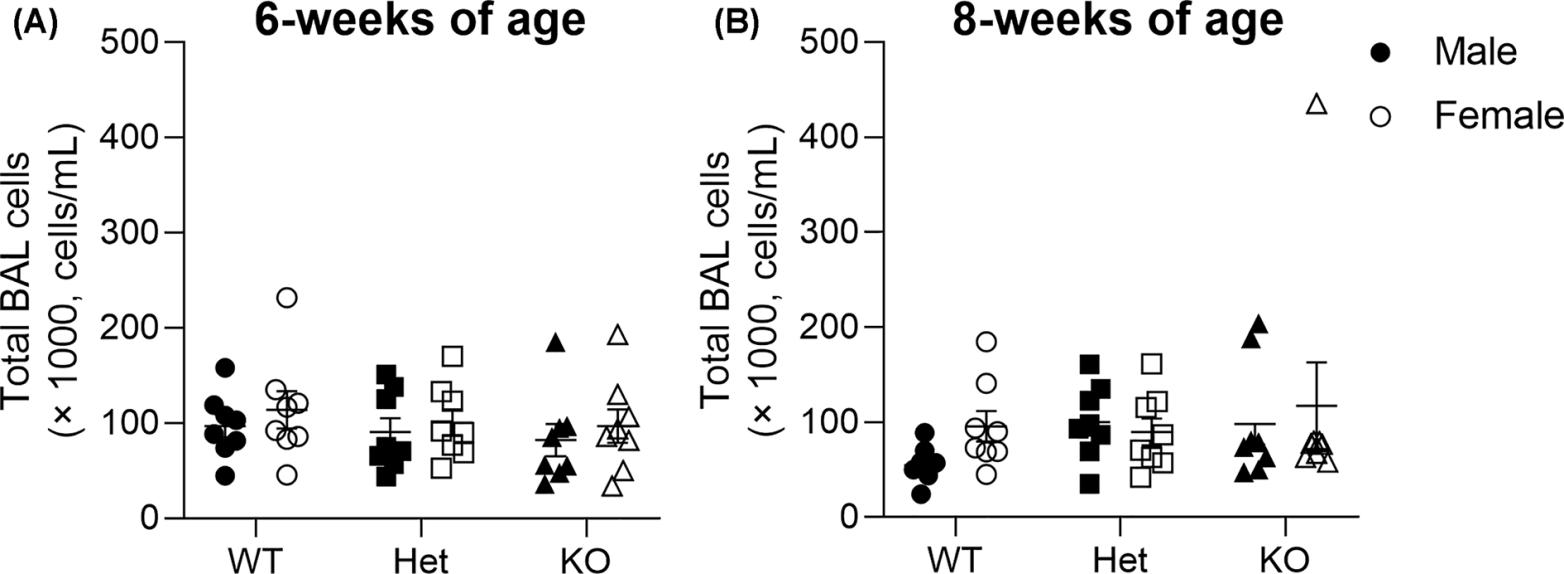
Total cell counts in BAL fluid Six weeks (**A**) and eight weeks ( **B**) of age. Data are presented as mean ± SEM. Closed circles represent WT males, open circles represent WT females, closed squares represent Het males, open squares represent Het females, closed triangles represent KO males, open triangles represent KO females. Six and eight weeks of age: WT, *n* = 8 males, *n* = 8 females; Het, *n* = 8 males, *n* = 8 females; KO, *n* = 8 males; *n* = 8 females. BAL, bronchoalveolar lavage; Het, Heterozygous; KO, knockout; WT, wild-type.

There were no genotypic differences in fasting basal glucose concentrations at either 6 (*P*=0.185, Figure 7A) or 8 weeks of age (*P*=0.807, Figure 7B). Comparison of glucose tolerance showed no genotypic differences between groups at 6 (*P*=0.974, Figure 7A,C) or 8 weeks of age (*P*=0.271, Figure 7B,D). At 6 weeks of age, males had a higher basal glucose than females (*P*<0.001, Figure 7A) and at both time points, males displayed an impaired glucose tolerance compared with females (sex, *P*<0.05, Figure 7C,D).

### Total inflammatory cell counts within BAL fluid

At 6 weeks of age, total inflammatory cell counts from BAL fluid did not vary between genotypes or sex (genotype, *P*=0.440; sex, *P*=0.293; Figure 8A). Similarly, there were no genotypic or sex differences in total inflammatory cell counts within the BAL fluid at 8 weeks of age (genotype, *P*=0.256; sex, *P*=0.286; Figure 8B).

### Metabolic profile

Plasma biomarker levels (C-peptide, gastric inhibitory peptide, glucagon, insulin, leptin, and peptide YY) were comparable between genotypes (genotype *P*>0.05; sex, *P*>0.05; Table 6). Plasma resistin was similar between genotypes but higher in females than males (genotype, *P*=0.711; sex, *P*=0.018; Table 6).

**Table 6:** Plasma biomarker concentrations

	WT		Het		KO	
Plasma biomarkers (pg/µl)	Male (*n*=4)	Female (*n*=5)	Male (*n*=3)	Female (*n*=5)	Male (*n*=3)	Female (*n*=3)
**C-peptide**	483 ± 121	807 ± 151	665 ± 135	685 ± 151	562 ± 108	693 ± 162
**Insulin**	1312 ± 195	1668 ± 318	1697 ± 271	1287 ± 349	1168 ± 177	1504 ± 206
**Gastric inhibitory polypeptide**	105 ± 17	153 ± 75	153 ± 24	155 ± 46	120 ± 12	108 ± 29
**Glucagon**	267 ± 60	265 ± 86	169 ± 85	196 ± 35	226 ± 73	180 ± 47
**Leptin**	896 ± 292	2042 ± 558	1055 ± 163	2087 ± 283	1949 ± 1249	1399 ± 79
**Peptide YY**	37 ± 13	39 ± 24	8 ± 8	79 ± 39	18 ± 13	47 ± 13
**Resistin**	26908 ± 1868†	37503 ± 2467	25725 ± 1728†	34591 ± 2451	32794 ± 4122†	30133 ± 1655

† denotes *P*<0.05 compared with females. Data are presented as mean ± SEM. Het, heterozygous; KO, knockout; WT, wild-type.

## Discussion

Airway-associated adipose tissue is metabolically active and its accumulation in overweight/obese subjects is correlated with wall thickness [3,4]. However, the genetic origin of airway-associated adipose tissue and its mechanistic role in airway remodelling and lung function impairment is unclear. After identifying *Kiss1*/*Kiss1r* signalling as a potential candidate for fatty airway remodelling, we investigated the role of *Kiss1*/*Kiss1r* signalling on airway adiposity and any associated pathophysiology using a KO mouse model. We found that the lack of *Kiss1*/*Kiss1r* signalling leads to the independent accumulation of airway-associated adipose tissue in the absence of body adiposity in prepubescent mice, indicating that airway adiposity has a strong genetic component. In adult *Kiss1r* KO mice, airway-associated adipose tissue continued to expand without affecting airway inflammation or lung function, which suggests that airway adiposity alone does not drive pathology but may instead integrate with existing disease or environmental factors in the context of comorbid asthma-obesity.

We demonstrate that *Kiss1* and *Kiss1r* genes are expressed within homogenised lung tissue from mice, consistent with findings on mouse ASM isolated after laser capture microdissection [19]. Expression of *Kiss1* was similar across genotypes and *Kiss1r* was only expressed in WT and Het mice, validating the use of the *Kiss1r* KO mouse model. After thorough examination on glucose handling there was no evidence of a diabetic condition which is observed in older (>10 weeks of age) *Kiss1r* KO mice [11,13]. Notably, diabetes is independently associated with asthma [20,21] and should be considered when examining the effects of obesity. Present results were, however, not affected by the confounding effects of diabetes in obesity.

At 6 weeks of age (prepubescence), we observed an increase in airway-associated adipose tissue in KO mice despite a lack of genotypic differences in body adiposity, glucose tolerance and systemic inflammation, as indicated by plasma biomarkers. These data support a new mechanism for airway-associated adipose tissue accumulation due to a genetic driver that is not affected by metabolic profile. The current observations differ from the relationship documented in our clinical study, where airway adiposity was associated with BMI [3]; although based on a relatively modest coefficient of determination, it was clear there were other contributing factors. Airway-associated adipose tissue is therefore at least partly decoupled from body adiposity. Similar to prepubescent KO mice, young adult KO mice demonstrated an increased accumulation of airway-associated adipose tissue with a greater proportion of both central and peripheral airways containing adipose tissue deposits. Female KO mice also displayed greater body weight and WAT deposition, which could give the false impression that body adiposity is contemporaneously related to airway-associated adipose tissue. Clearly, airway-associated adipose tissue expansion manifested differently and began accumulating before the increase in body adiposity.

The severity of obesity in young adult KO mice can be considered relatively mild in comparison with other models. Caloric overconsumption can increase body weight by up to ∼30% within 20 weeks of feeding compared with control counterparts [22]. Mice fed a high-fat diet achieved an average body weight of 43 g at 22 weeks of age, which increased to 50 g by 30 weeks of age [23]. In our study, female KO mice at 8 weeks of age had an average body weight of 23 g, representing a ∼7% increase in size over WT mice. Prior metabolic studies using this model show that by 20 weeks of age, female KO mice are ∼32% larger than WT counterparts [13].

Increased airway adiposity may exacerbate inflammation through release of pro-inflammatory adipokines [4]. However, there was no increase in inflammatory cells within the BAL fluid of mice expressing greater airway-associated adipose tissue content. We have demonstrated that airway-associated adipose tissue accumulation alone is not sufficient to stimulate infiltration of immune cells within the airway wall. Previous findings describe the positive association between airway-associated adipose tissue accumulation and neutrophilic airway inflammation in control and non-fatal subjects with asthma, and eosinophilic inflammation in subjects who died of asthma [3]. The immune response in comorbid asthma-obesity is notoriously heterogenous where obese patients with asthma are further categorised into phenotypes depending on the degree of atopy [24]. Superimposing allergy [25] onto our genetic model of obesity may lead to an infiltration of immune cells and upregulation of inflammatory pathways.

There was some indication of a change in airway wall structure in young adult female KO mice who had a greater ASM layer thickness (∼10%) in peripheral airways compared with female Het mice, with an increase in WA_i_ (which includes the ASM layer) only observed when compared with WT. Remodelling of ASM varies within and between patients with asthma, with a relatively small number of cases (<20%) identified as having ASM remodelling in only peripheral airways [26]. There is, however, emerging data to support a link between *Kiss1r* biology and ASM remodelling [7]. Expression of *Kiss1r* is reduced in ASM from asthmatic subjects compared with non-asthmatic subjects, and in cell culture *Kiss1r* antagonism increases ASM proliferation [7]. In an allergic mouse model, intranasal instillation of *Kiss1* reduced ASM thickness [19]. The interrelationship between *Kiss1*/*Kiss1r* signalling, airway-associated adipose tissue accumulation and ASM remodelling is an area of research that should receive greater focus in the future.

Any change in lung function between genotypes may reflect differences in body weight, airway-associated adipose tissue, ASM layer thickness or other biological factors developed as a result of genetic manipulation. Given the modest change in body weight, functional assessment was unlikely to be affected by compression of the chest by fat deposition or induction of systemic/airway inflammation through adipokine release [27,28]. Remodelling of the ASM layer was relatively mild, peripherally limited and was not accompanied by any change in bronchoconstrictor response. The most prominent change was the ∼50% increase in airway-associated adipose tissue in *Kiss1r* KO mice; despite this substantial change in airway adiposity there was no simultaneous effect on baseline or post-bronchial challenge *R*_aw_, *G* or *H*. These data suggest that airway-associated adipose tissue expansion alone, at least up until young adulthood, does not directly impair lung function and may instead act as a contributing factor to disease severity, in much the same way as proposed interactive effects of inflammation and remodelling [14], or generalised body fat in a patient with asthma.

Airway-associated adipose tissue was expanded in both male and female KO mice, in contrast to WAT, which was only increased in adult female KO. That is, between KO mice, airway-associated adipose tissue relative to body adiposity is higher in males than in females, in broad agreement with clinical findings where airway-associated adipose tissue was greater in males than females at a given BMI [3]. Female sex hormones, particularly oestrogen, have been shown to play a protective role in reducing the deposition and inflammatory phenotype of adipose tissue [29,30]. For example, reduced oestrogen production in ovariectomised female mice increases the accumulation of body adiposity compared with intact littermates [13]. The same sex hormone also impacts the functional behaviour and/or structure of the airway; activation of oestrogen receptors in mice reduces mixed allergen-induced bronchoconstriction and remodelling, specifically collagen and α-smooth muscle actin expression [31]. With the above in mind, the literature supports an interesting interaction between sex, obesity, and lung disease, although despite differences in both body and airway adiposity relative to body weight, in the present study there was no effect of sex on other airway wall dimensions, or on lung mechanics.

Sex differences independent of *Kiss1*/*Kiss1r* signalling are also noted. Irrespective of genotype, prepubertal male mice had greater abdominal circumference and adiposity, accompanied by an impaired glucose tolerance, consistent with previous findings [32]. However, while such abnormalities in metabolic function are typically accompanied by inflammatory changes [33], there was no such evidence in prepubertal male mice. Instead, serum resistin (a pro-inflammatory adipokine) was elevated in females compared with males, with no changes to airway inflammation. Despite an increase in Δ*G* and Δ*H*, females did not demonstrate bronchoconstrictor response to MCh. Prepubertal males had a greater ASM thickness throughout the lung than females, which did not affect lung function. The above differences between males and females were transient; by 8 weeks of age, airway structure, adiposity and lung function for the most part were comparable.

Does *Kiss1*/*Kiss1r* signalling within adipose tissue modify airway adiposity? While expression of *Kiss1* and *Kiss1r* in WAT has been established [34], the specific pathways modulating local lipid metabolism are unknown. Activation of *Kiss1*/*Kiss1r* signalling in mature adipocytes increases lipolysis and impairs both differentiation and adipogenesis in preadipocytes [35]. Expression of *Kiss1* and *Kiss1r* is reduced in WAT from obese mice compared with healthy weight counterparts and is restored by weight loss [36]. In rats fed a high-fat diet, there was a reduction in *Kiss1* expression within WAT [34], suggesting that diet-induced obesity may disrupt *Kiss1*/*Kiss1r* signalling. Together these findings support abnormalities in *Kiss1*/*Kiss1r* signalling and subsequent changes to lipolysis and adipogenesis as one possible driver of airway adiposity.

To conclude, we have demonstrated that airway-associated adipose tissue can accumulate in the absence of body adiposity and identified *Kiss1*/*Kiss1r* signalling as a genetic origin. We previously hypothesized that airway-associated adipose tissue may impact airway structure–function in comorbid asthma-obesity [37]; the present study re-emphasizes airway-associated adipose tissue as another feature of airway remodelling that may become important in later life with inevitable exposure to environmental triggers. The full life impact of airway-associated adipose tissue should continue to be examined as well as potential therapies beyond weight loss that may optimize respiratory health.

## Clinical perspectives

- Airway-associated adipose tissue has been shown to accumulate with BMI and may contribute to greater asthma severity in obese patients. Whether airway adiposity has a genetic predisposition and how it impacts airway structure–function and inflammation is unknown.
- The *Kiss1*/*Kiss1r* signalling pathway is a genetic origin for airway adiposity. Airway-associated adipose tissue can accumulate independent of body adiposity.
- Findings emphasise airway-associated adipose tissue expansion as another feature of airway remodelling. Targeting airway-associated adipose tissue in patients where it is abundant may be effective in improving respiratory function that has been compromised by environmental exposures.

## Data Availability

The data that support the findings of this study are available from the corresponding author upon reasonable request.

## Open Access

Open access for this article was enabled by the participation of The University of Western Australia in an all-inclusive *Read & Publish* agreement with Portland Press and the Biochemical Society under a transformative agreement with CAUL.

## Abbreviations

ASM: airway smooth muscle
AUC: area under the curve
BAL: bronchoalveolar lavage
BMI: body mass index
*G*: airway tissue damping
*H*: airway tissue elastance
H&E: hematoxylin and eosin
Het: hetereozygous
*Kiss1*: kisspeptin
*Kiss1r*: kisspeptin receptor
KO: knockout
MCh: methacholine
*P*_bm_: perimeter of the basement membrane
PPIA: peptidylprolyl isomerase A
qPCR: quantitative real-time PCR
*R*_aw_: airway resistance
SDHA: succinate dehydrogenase
TBP: TATA-binding protein
WA_i_: inner wall area
WA_o_: outer wall area
WAT: white adipose tissue
WA_t_: total wall area
WT: wild-type

## References

[1] Bergeron C., Tulic M.K. and Hamid Q. (2010) Airway remodelling in asthma: from benchside to clinical practice. Can. Respir. J. 17, e85–e93 10.1155/2010/318029 20808979 PMC2933777

[2] Peters U., Dixon A.E. and Forno E. (2018) Obesity and asthma. J. Allergy Clin. Immunol. 141, 1169–1179 10.1016/j.jaci.2018.02.004 29627041 PMC5973542

[3] Elliot J.G., Donovan G.M., Wang K.C.W., Green F.H.Y., James A.L. and Noble P.B. (2019) Fatty airways: implications for obstructive disease. Eur. Respir. J. 54, 1900857 10.1183/13993003.00857-2019 31624112

[4] Wang C.J., Noble P.B., Elliot J.G., Choi Y.S., James A.L. and Wang K.C.W. (2023) Distribution, composition, and activity of airway-associated adipose tissue in the porcine lung. Am. J. Physiol. Lung Cell. Mol. Physiol. 324, L179–L189 10.1152/ajplung.00288.2022 36445102

[5] Loos R.J.F. and Yeo G.S.H. (2022) The genetics of obesity: from discovery to biology. Nat. Rev. Genet. 23, 120–133 10.1038/s41576-021-00414-z 34556834 PMC8459824

[6] Willis-Owen S.A.G., Cookson W.O.C. and Moffatt M.F. (2018) The genetics and genomics of asthma. Annu. Rev. Genomics Hum. Genet. 19, 223–246 10.1146/annurev-genom-083117-021651 30169121

[7] Borkar N.A., Ambhore N.S., Kalidhindi R.S.R., Pabelick C.M., Prakash Y.S. and Sathish V. (2022) Kisspeptins inhibit human airway smooth muscle proliferation. JCI Insight 7, e152762 10.1172/jci.insight.152762 35420998 PMC9220928

[8] Lei Z., Bai X., Ma J. and Yu Q. (2019) Kisspeptin-13 inhibits bleomycin-induced pulmonary fibrosis through GPR54 in mice. Mol. Med. Rep. 20, 1049–1056 10.3892/mmr.2019.10341 31173221 PMC6625411

[9] Tolson K.P., Marooki N., De Bond J.P., Walenta E., Stephens S.B.Z., Liaw R.B. et al. (2020) Conditional knockout of kisspeptin signaling in brown adipose tissue increases metabolic rate and body temperature and lowers body weight. FASEB J. 34, 107–121 10.1096/fj.201901600R 31914628 PMC7202476

[10] Hudson A.D. and Kauffman A.S. (2022) Metabolic actions of kisspeptin signaling: Effects on body weight, energy expenditure, and feeding. Pharmacol. Ther. 231, 107974 10.1016/j.pharmthera.2021.107974 34530008 PMC8884343

[11] Tolson K.P., Garcia C., Yen S., Simonds S., Stefanidis A., Lawrence A. et al. (2014) Impaired kisspeptin signaling decreases metabolism and promotes glucose intolerance and obesity. J. Clin. Invest. 124, 3075–3079 10.1172/JCI71075 24937427 PMC4071390

[12] Han S.K., Gottsch M.L., Lee K.J., Popa S.M., Smith J.T., Jakawich S.K. et al. (2005) Activation of gonadotropin-releasing hormone neurons by kisspeptin as a neuroendocrine switch for the onset of puberty. J. Neurosci. 25, 11349–11356 10.1523/JNEUROSCI.3328-05.2005 16339030 PMC6725899

[13] Tolson K.P., Garcia C., Delgado I., Marooki N. and Kauffman A.S. (2016) Metabolism and energy expenditure, but not feeding or glucose tolerance, are impaired in young kiss1r KO female mice. Endocrinology 157, 4192–4199 10.1210/en.2016-1501 27649089 PMC5086529

[14] Wang K.C.W., Le Cras T.D., Larcombe A.N., Zosky G.R., Elliot J.G., James A.L. et al. (2018) Independent and combined effects of airway remodelling and allergy on airway responsiveness. Clin. Sci. (Lond.) 132, 327–338 10.1042/CS20171386 29269381

[15] Donovan G.M., Wang K.C.W., Shamsuddin D., Mann T.S., Henry P.J., Larcombe A.N. et al. (2020) Pharmacological ablation of the airway smooth muscle layer - Mathematical predictions of functional improvement in asthma. Physiol. Rep. 8, e14451 10.14814/phy2.14451 32533641 PMC7292900

[16] Smoothy J., Larcombe A.N., Chivers E.K., Matthews V.B. and Gorman S. (2019) Maternal high fat diet compromises survival and modulates lung development of offspring, and impairs lung function of dams (female mice). Respir. Res. 20, 21 10.1186/s12931-019-0976-3 30700289 PMC6354360

[17] Looi K., Kicic A., Noble P.B. and Wang K.C.W. (2021) Intrauterine growth restriction predisposes to airway inflammation without disruption of epithelial integrity in postnatal male mice. J. Dev. Orig. Health Dis. 12, 496–504 10.1017/S2040174420000744 32799948

[18] Van Hoecke L., Job E.R., Saelens X. and Roose K. (2017) Bronchoalveolar lavage of murine lungs to analyze inflammatory cell infiltration. J. Vis. Exp. 123 55398 10.3791/55398 28518083 PMC5607888

[19] Borkar N.A., Ambhore N.S., Balraj P., Ramakrishnan Y.S. and Sathish V. (2023) Kisspeptin regulates airway hyperresponsiveness and remodeling in a mouse model of asthma. J. Pathol. 260, 339–352 10.1002/path.6086 37171283 PMC10759912

[20] Nie Z., Jacoby D.B. and Fryer A.D. (2014) Hyperinsulinemia potentiates airway responsiveness to parasympathetic nerve stimulation in obese rats. Am. J. Respir. Cell Mol. Biol. 51, 251–261 10.1165/rcmb.2013-0452OC 24605871 PMC4148040

[21] Brumpton B.M., Camargo C.A. Jr, Romundstad P.R., Langhammer A., Chen Y. and Mai X.M. (2013) Metabolic syndrome and incidence of asthma in adults: the HUNT study. Eur. Respir. J. 42, 1495–1502 10.1183/09031936.00046013 23845717

[22] Speakman J., Hambly C., Mitchell S. and Krol E. (2007) Animal models of obesity. Obes. Rev. 8, 55–61 10.1111/j.1467-789X.2007.00319.x 17316303

[23] Johnston R.A., Theman T.A., Lu F.L., Terry R.D., Williams E.S. and Shore S.A. (2008) Diet-induced obesity causes innate airway hyperresponsiveness to methacholine and enhances ozone-induced pulmonary inflammation. J. Appl. Physiol. (1985) 104, 1727–1735 10.1152/japplphysiol.00075.2008 18323466

[24] Holguin F., Bleecker E.R., Busse W.W., Calhoun W.J., Castro M., Erzurum S.C. et al. (2011) Obesity and asthma: an association modified by age of asthma onset. J. Allergy Clin. Immunol. 127, 1486.e2–1493.e2 10.1016/j.jaci.2011.03.036 21624618 PMC3128802

[25] Carroll O.R., Pillar A.L., Brown A.C., Feng M., Chen H. and Donovan C. (2023) Advances in respiratory physiology in mouse models of experimental asthma. Front Physiol. 14, 1099719 10.3389/fphys.2023.1099719 37008013 PMC10060990

[26] James A.L., Donovan G.M., Green F.H.Y., Mauad T., Abramson M.J., Cairncross A. et al. (2023) Heterogeneity of airway smooth muscle remodeling in asthma. Am. J. Respir. Crit. Care Med. 207, 452–460 10.1164/rccm.202111-2634OC 36399661

[27] Jones R.L. and Nzekwu M.M. (2006) The effects of body mass index on lung volumes. Chest 130, 827–833 10.1378/chest.130.3.827 16963682

[28] Pinkerton J.W., Kim R.Y., Brown A.C., Rae B.E., Donovan C., Mayall J.R. et al. (2021) Relationship between type 2 cytokine and inflammasome responses in obesity-associated asthma. J. Allergy Clin. Immunol. 149, 1270–1280, S0091-6749, 01525-6 10.1016/j.jaci.2021.10.003 34678326

[29] Dakin R.S., Walker B.R., Seckl J.R., Hadoke P.W. and Drake A.J. (2015) Estrogens protect male mice from obesity complications and influence glucocorticoid metabolism. Int. J. Obes. (Lond.) 39, 1539–1547 10.1038/ijo.2015.102 26032810 PMC4564952

[30] Imano N., Shojima K., Tamaki K. and Shinmura K. (2023) Estrogen contributes to the sex difference in the occurrence of senescence-related T cells during the development of visceral adipose tissue inflammation. Am. J. Physiol. Heart Circ. Physiol. 324, H662–H674 10.1152/ajpheart.00469.2022 36930655

[31] Ambhore N.S., Kalidhindi R.S.R., Loganathan J. and Sathish V. (2019) Role of differential estrogen receptor activation in airway hyperreactivity and remodeling in a murine model of asthma. Am. J. Respir. Cell Mol. Biol. 61, 469–480 10.1165/rcmb.2018-0321OC 30958966 PMC6775953

[32] Atamni H.J., Mott R., Soller M. and Iraqi F.A. (2016) High-fat-diet induced development of increased fasting glucose levels and impaired response to intraperitoneal glucose challenge in the collaborative cross mouse genetic reference population. BMC Genet. 17, 10 10.1186/s12863-015-0321-x 26728312 PMC4700737

[33] Tsalamandris S., Antonopoulos A.S., Oikonomou E., Papamikroulis G.A., Vogiatzi G., Papaioannou S. et al. (2019) The role of inflammation in diabetes: Current concepts and future perspectives. Eur. Cardiol. 14, 50–59 10.15420/ecr.2018.33.1 31131037 PMC6523054

[34] Brown R.E., Imran S.A., Ur E. and Wilkinson M. (2008) Kiss-1 mRNA in adipose tissue is regulated by sex hormones and food intake. Mol. Cell. Endocrinol. 281, 64–72 10.1016/j.mce.2007.10.011 18069123

[35] Pruszynska-Oszmalek E., Kolodziejski P.A., Sassek M. and Sliwowska J.H. (2017) Kisspeptin-10 inhibits proliferation and regulates lipolysis and lipogenesis processes in 3T3-L1 cells and isolated rat adipocytes. Endocrine 56, 54–64 10.1007/s12020-017-1248-y 28194651

[36] Gomes V.C.L., Beckers K.F., Crissman K.R., Landry C.A., Flanagan J.P., Awad R.M. et al. (2023) Sexually dimorphic pubertal development and adipose tissue kisspeptin dysregulation in the obese and preeclamptic-like BPH/5 mouse model offspring. Front Physiol. 14, 1070426 10.3389/fphys.2023.1070426 37035685 PMC10076539

[37] Wang C.J., Noble P.B., Elliot J.G., James A.L. and Wang K.C.W. (2022) From beneath the skin to the airway wall: understanding the pathological role of adipose tissue in comorbid asthma-obesity. Compr. Physiol. 13, 4321–4353 10.1002/cphy.c220011 36715283

